# Pyrroloquinoline quinone (PQQ) alleviated sepsis-induced acute liver injury, inflammation, oxidative stress and cell apoptosis by downregulating CUL3 expression

**DOI:** 10.1080/21655979.2021.1935136

**Published:** 2021-07-06

**Authors:** Yanhong Wu, Meiling Zhao, Zhaoheng Lin

**Affiliations:** a Department of Critical Care Medicine, Hunan Provincial People’s Hospital (The First Affiliated Hospital of Hunan Normal University), Changsha, Hunan Province, China; b Department of Critical Care Medicine, Zibo Central Hospital, Zibo, Shandong Province, China; c Department of Critical Care Medicine, The People’s Hospital of Xishuangbanna Dai Nationality Autonomous Prefecture, Jinghong, Yunnan Province, China

**Keywords:** PQQ, sepsis, acute liver injury, CUL3, inflammation, oxidative stress

## Abstract

PQQ has anti-inflammatory and anti-oxidant effects. PQQ can relieve high glucose-induced renal cell damage by suppressing Keap1 expression. Keap1 can interact with CUL3. Upregulation of CUL3 facilitates the apoptosis of LPS-induced podocytes. Based on knowledge above, this current work was designed to explore the role of PQQ in sepsis and determine the molecular function of CUL3 in the pathogenesis of sepsis. Rats received CLP surgery to establish sepsis models in vivo. Kupffer cells were pretreated with PQQ (10, 50 and 100 nmol/L) for 2 h and then treated with 100 ng/mL LPS for 24 h, simulating sepsis-induced acute liver injury in vitro. H&E staining was performed to evaluate liver injury of SD rats. Levels of inflammatory factors and oxidative stress markers were detected to assess inflammatory response and oxidative stress. Moreover, TUNEL staining, flow cytometric analysis and western blot were applied to determine cell apoptosis. It was confirmed that PQQ treatment relieved acute liver injury, inflammatory and oxidative stress damage and apoptosis of liver tissue cells in sepsis rats. In addition, PQQ therapy could alleviate inflammation, oxidative stress and apoptosis in LPS-induced Kupffer cells. Notably, LPS stimulation enhanced CUL3 expression and PQQ repressed CUL3 expression in Kupffer cells suffered from LPS. Overall, CUL3 overexpression weakened the remission effects of PQQ on LPS-induced inflammatory and oxidative damage and apoptosis of Kupffer cells. Mechanistically, PQQ treatment may mitigate sepsis-induced acute liver injury through downregulating CUL3 expression.

## Graphical Abstract

**Figure:**
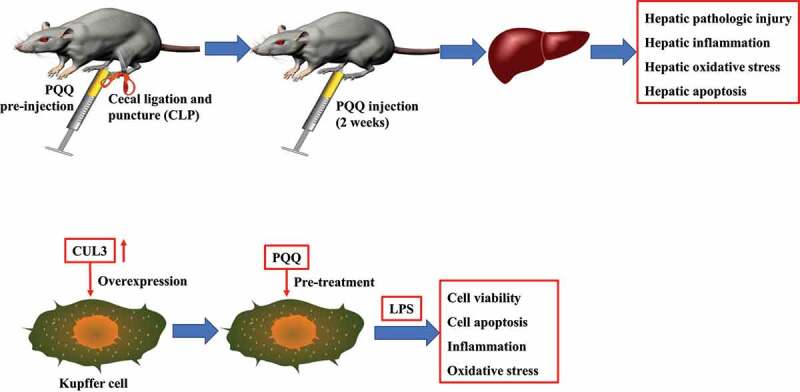

## Introduction

The weak ability of body in regulating infection will induce the occurrence and development of systemic inflammatory response, ultimately resulting in sepsis [1–3]. In addition, sepsis can also lead to multiple organ dysfunction and damage [4]. Till now, the global morbidity and mortality of sepsis are still increasing year by year [5,6]. Recently, accompanied by the development of antibiotics and new therapies, the mortality of patients with sepsis has declined to a certain degree. However, the mortality of septic shock caused by sepsis remains a very high level so far [7,8]. Hence, elucidation of the molecular mechanisms underlying sepsis progression and identifying novel therapeutic drugs are critical for sepsis clinically.

Pyrroloquinoline quinone (PQQ) is a redox cycle coenzyme which consists of nicotinamide and flavonoid. PQQ has been proven to widely exist in various foods (vegetables, fruits, milk, etc.) [9]. A research has revealed that PQQ can prevent the occurrence and development of knee osteoarthritis by relieving oxidative stress [10]. Furthermore, several studies suggest that PQQ can alleviate inflammatory responses in multiple tissues [11,12]. For example, PQQ can ameliorate LPS-induced neuroinflammation of mice [13]. PQQ can also delay the progression of rheumatoid arthritis by repressing inflammatory response [14].

In addition, PQQ can relieve oxidative stress induced inflammatory injury of human renal tubular epithelial cells by suppressing Keap1 expression [15]. Importantly, database (String, https://string-db.org/) displays that Keap1 targets and interacts with cullin 3 (CUL3). Besides, it is confirmed that CUL3 is elevated in LPS-induced mouse podocytes and miR-15a-5p inhibitor facilitates the apoptosis of LPS-induced podocytes by upregulating CUL3 expression [16]. Moreover, Liao et al. [17] verifies that Keap 1 and CUL3 are upregulated in lungs of LPS-induced rats. Knowledge above prompts that PQQ may function in multiple types of organs and tissues by repressing CUL3.

Here, we reported the protective role of PQQ against sepsis-induced acute liver injury in both in vivo and in vitro models. PQQ treatment mitigated acute liver injury, inflammatory response, oxidative stress and cell apoptosis in sepsis rats. In addition, PQQ could also alleviate inflammation, oxidative stress and cell apoptosis of LPS-stimulated Kupffer cells. Mechanically, results delineated that PQQ may ameliorate sepsis-induced acute liver injury through suppressing CUL3 expression. To conclude, the current study will develop novel insights into the molecular function of CUL3 in the pathogenesis of sepsis and highlight the role of PQQ in sepsis therapies.

## Materials and methods

### Establishment of sepsis rat models

Twenty SPF Sprague Dawley (SD) rats were purchased from Qinglongshan animal breeding farm (Nanjing, Jiangsu, China). SD rats were randomly divided into four groups (Control, PQQ, CLP and PQQ+CLP). Rats received CLP surgery to establish sepsis models in vivo [1]. Briefly, rats were injected with 10% chloral hydrate intraperitoneally to induce anesthesia. Under sterile surgical conditions, a 1.0-cm incision was made to expose the cecum. Then, 4/0 surgical silk was used to ligate the cecum at a distance of approximately 1.5 cm from the end of the cecum. The cecum was then punctured twice with a 24-gauge needle and fecal contents were expelled by squeezing cecum. The bowel was then placed in the peritoneal cavity and the abdominal cavity was closed. Rats were intraperitoneally administrated with PQQ (10 mg/kg; Sigma-Aldrich, St. Louis, MO, USA) 1 h before surgery and continuously administrated with PQQ (10 mg/kg) for 2 weeks after Cecal ligation and puncture (CLP) surgery. After that, rats were sacrificed after anesthesia and liver tissues and peripheral blood were collected for the subsequent detection. Body weights of rats were recorded before sacrifice.

### Cell culture

Kupffer cells were obtained from ATCC (Manassas, VA, USA) and cultured in Dulbecco’s modified Eagle’s medium (DMEM) (HyClone, South Logan, UT, USA) supplemented with 10% fetal bovine serum (Gibco, Grand Island, NY, USA). Kupffer cells were maintained at 37°C in a humidified environment containing 5% CO_2_.

### Cell treatment

Kupffer cells were pretreated with PQQ (10, 50 and 100 nmol/L) for 2 h and then treated with 100 ng/mL LPS for 24 h.

### Cell transfection

For transfection, CUL3 was cloned into pcDNA 3.1 (Ov-CUL3) and the empty vector (Ov-NC) served as negative control. All the vectors were designed by Genechem (Shanghai, China). Briefly, cells were grown up to 90% confluence and then transfected with lentivirus diluted with Opti-MEM (Gibco, Grand Island, NY, USA) serum-free medium using Lipofectamine 3000 reagent (Invitrogen, Carlsbad, CA, USA) following the manufacturer’s instructions.

### Hemotoxylin and eosin (H&E) staining

The liver tissues of SD rats were fixed in 4% formaldehyde, embedded with paraffin and cut into sections (5 µm) for H&E staining. Next, liver sections were stained with hematoxylin and eosin (Thermo Fisher Scientific, Waltham, MA, USA). Finally, liver sections were observed and photographed under the light microscope (Olympus, Tokyo, Japan).

### Detection of oxidative stress markers

The expressions of oxidative stress markers malondialdehyde (MDA), glutathione (GSH) were determined with the commercial kits (Nanjingjiancheng company, Nanjing, China).

### ELISA assays

Rats IL-1β ELISA kit (Abcam, Cambridge, MA, USA, ab100768), rats TNF-α ELISA kit (Abcam, Cambridge, MA, USA, ab100785), rats IL-6 ELISA kit (Abcam, Cambridge, MA, USA, ab100772), rats ALT ELISA kit (Abcam, Cambridge, MA, USA, ab234579), rats AST ELISA kit (Abcam, Cambridge, MA, USA, ab263883) and rats ALP ELISA kit (Abcam, Cambridge, MA, USA, ab267583) were used for ELISA assays in this research. All operations followed the manufacturer’s instruction.

### TdT-mediated dUTP nick-end labeling (TUNEL) staining

Apoptosis of liver tissues was detected using TUNEL staining kit (Roche, Basel, Switzerland). Briefly, liver tissues of SD rats were cut into sections. Next, these sections were deparaffinized. After that, the sections were permeabilized with 0.2% Triton X-100 for 10 min at room temperature and then stained with 50 μM TUNEL reagent for 1 h at 37°C in the dark. Then, 4ʹ-6-diamino-2-phenylindole (DAPI; Sigma-Aldrich, St. Louis, MO, USA) was used to stain the cell nuclei for 20 min in the dark. Finally, the positive cells were visualized and counted under a fluorescence microscope (Leica, Wetzlar, Germany).

### Apoptosis assays

In brief, Kupffer cells following designed treatment were harvested for cell apoptosis analysis. Kupffer cells were double-stained with FITC-conjugated Annexin V and PI (Beyotime, Shanghai, China) for 15 min in the dark. The percentage of apoptotic cells was analyzed on the flow cytometry (Thermo Fisher Scientific, Waltham, MA, China).

### Counting Kit-8 (CCK-8) assays

Kupffer cells were plated into the 96 well plates (2 × 10^3^ cells/holes). After cell attachment, cells were pretreated with PQQ (10, 50 and 100 nmol/L) for 2 h and then treated with 100 ng/mL LPS for 24 h. After that, CCK-8 solution (Beyotime, Shanghai, China) was added into Kupffer cells for another 4 h incubation at 37 ℃. Finally, the absorbance of each well was measured at 450 nm with the spectrophotometer (Thermo Fisher Scientific, Waltham, MA, USA).

### Real-time quantitative polymerase chain reaction (RT-qPCR)

Total RNA was extracted with the Trizol reagent (Thermo Fisher Scientific, Waltham, MA, USA). Next, RNA was reverse transcribed into cDNA by the reverse transcription kit (Roche, Basel, Switzerland). The SYBE Green (Thermo Fisher Scientific, Waltham, MA, USA) was applied for the fluorescence indicator in this assay. Then, cDNA was amplified with ABI 7500 system (Applied Biosystems, Foster City, CA, USA). The relative expression of target genes was calculated using 2^−∆∆Ct^ method. The primers used in this research were IL-1β forward primer: 5'-GCCATGGACAAGCTGAGGAAG-3' reverse primer: 5'-GTGCTGATGTACCAGTTGGG-3' IL-6 forward primer: 5'-GGCCCTTGCTTTCTCTTCG-3' reverse primer 5'-ATAATAAAGTTTTGATTATGT-3' TNF-α forward primer: 5'-GGATCTCAAAGACAACCAAC-3' reverse primer: 5'-ACAGAGCAATGACTCCAAAG-3' CUL3 forward primer: 5'-GATGAGTTCAGGCAACATC-3' reverse primer: 5'-ATGTCTTGGTGCTGGTGG-3'.

### Western blotting assays

Total proteins from liver tissues and Kupffer cells were extracted with RIPA lysis buffer (Beyotime, Shanghai, China). Next, the concentration of protein samples was determined using BCA methods. Equal amount of protein samples was separated by 10% SDS-PAGE gel (Beyotime, Shanghai, China) and transferred to PVDF membranes (Thermo Fisher Scientific, Waltham, MA, China). Subsequently, PVDF membranes were blocked with 5% skim milk powder and incubated with primary antibodies at 4°C overnight. The primary antibodies used in this research were Bcl-2 (Abcam, Cambridge, MA, USA, ab182858), Bax (Abcam, Cambridge, MA, USA, ab32503), Cleaved caspase-3 (Abcam, Cambridge, MA, USA, ab2302), Caspase-3 (Abcam, Cambridge, MA, USA, ab13847), Cleaved caspase-9 (Abcam, Cambridge, MA, USA, ab2324), Caspase-9 (Abcam, Cambridge, MA, USA, ab202068) and CUL3 (Abcam, Cambridge, MA, USA, ab108407). On the second day, PVDF membranes were washed twice with PBST and then incubated with secondary antibodies for 2 h at room temperature. Finally, protein bands were developed with chemiluminescence reagents (Millipore, Billerica, MA, USA) and analyzed using Image Lab.

### Statistical analysis

Experimental data in this research was analyzed using Graphpad Prism 7.0 (GraphPad Software Inc., La Jolla, CA, USA) and displayed as mean ± SD. One-way analysis of variance followed by Tukey’s post hoc test was performed to determine the differences among multiple groups. Differences between diverse groups were considered as remarkable when values of p were less than 0.05. All the experiments in this study were repeated for three times. * p < 0.05, **p < 0.01, ***p < 0.001.

## Results

### PQQ treatment relieved sepsis-induced acute liver injury

**Figure 1.**
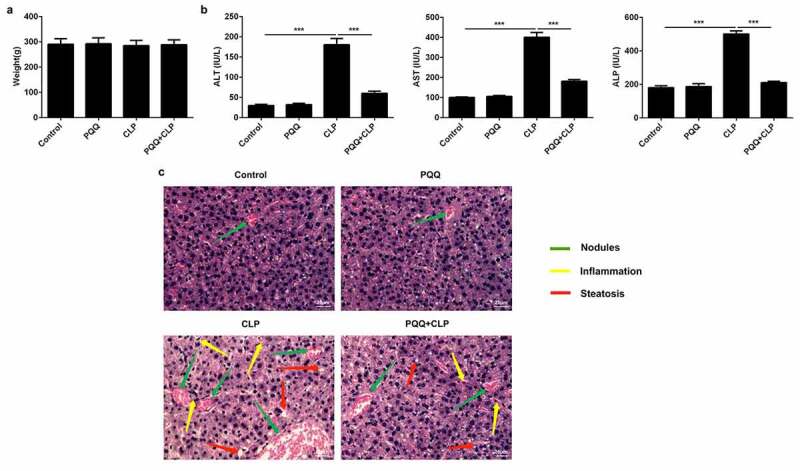
PQQ treatment relieved sepsis-induced acute liver injury. SD rats received CLP surgery to establish in vivo sepsis models. (a) Body weights of SD rats. (b) ALT, AST and ALP levels in the serum of SD rats. (c) HE staining of liver tissues

SD rats received CLP surgery to establish in vivo sepsis models. It was seen that the occurrence of sepsis had no obvious influence on the body weights of rats (Figure 1(a)). ALT, AST and ALP levels were enhanced in the serum of sepsis rats. PQQ treatment visibly declined ALT, AST and ALP levels, suggesting that PQQ may have the potential to protect against acute liver injury of sepsis rats (Figure 1(b)). In addition, results of HE staining discovered that sepsis induced the formation of nodules in liver tissues and promoted inflammatory response. Furthermore, PQQ therapy decreased the number of nodules and alleviated inflammation in sepsis rats (Figure 1(c)).

### PQQ treatment alleviated sepsis-induced inflammation and oxidative stress

**Figure 2.**
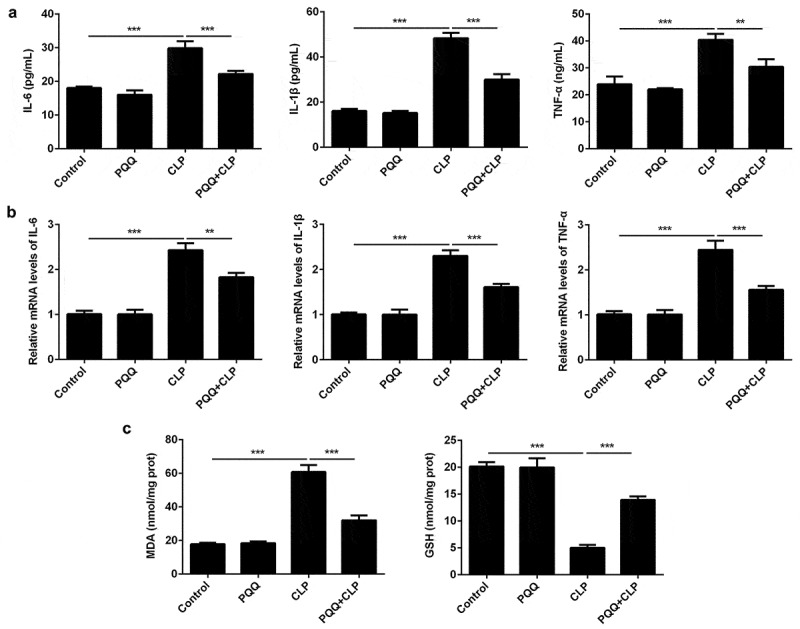
PQQ treatment alleviated sepsis-induced inflammation and oxidative stress. (a) Levels of IL-6, IL-1β and TNF-α in the serum of SD rats were detected using ELISA methods. (b) Expressions of IL-6, IL-1β and TNF-α in liver tissues of SD rats were detected by RT-qPCR assay. (c) The production of MDA and GSH in liver tissues of SD rats was detected with the commercial kits

Considering the well-known knowledge that inflammation and oxidative stress are closely associated with sepsis, levels of inflammatory factors and oxidative stress markers were assessed to evaluate the influence of PQQ on inflammatory response and oxidative stress in sepsis rats. Levels of inflammatory factors (IL-6, IL-1β and TNF-α) both in the serum and liver tissues of sepsis rats were significantly upregulated and PQQ treatment partly reversed the promotion of inflammatory response (Figure 2(a, b)). Furthermore, increased MDA expression and decreased GSH expression in liver tissues of sepsis rats were rescued by PQQ therapy, indicating that PQQ could relieve sepsis-induced oxidative stress (Figure 2(c)).

### PQQ treatment mitigated sepsis-induced cell apoptosis of liver tissues

**Figure 3.**
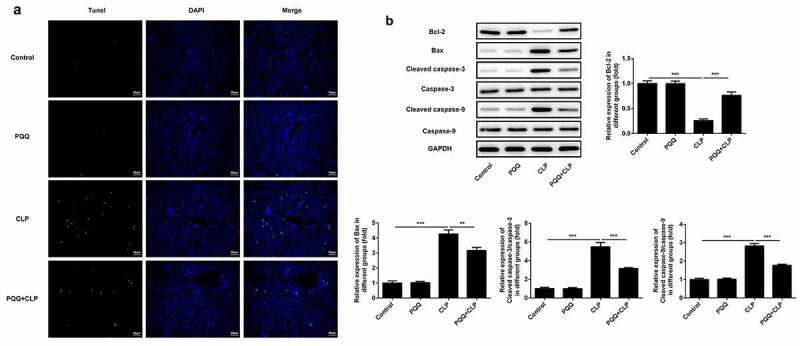
PQQ treatment mitigated sepsis-induced cell apoptosis of liver tissues. (a) Apoptosis of liver tissue cells was detected with TUNEL staining. (b) Expressions of Bcl-2, Bax, Cleaved caspase3 and Cleaved caspase9 were determined using western blotting analysis

TUNEL staining was performed to detect the apoptosis of liver tissues. Apoptosis of liver tissue cells was aggravated after the occurrence of sepsis and PQQ treatment relieved the apoptosis of liver tissue cells (Figure 3(a)). Furthermore, expressions of apoptosis-related proteins were determined using western blotting analysis. It was observed that expressions of Bax, Cleaved caspase-3 and Cleaved caspase-9 were enhanced and Bcl-2 expression was decreased after the occurrence of sepsis. Then, PQQ treatment rescued the reduction of Bcl-2 expression and elevation of Bax, Cleaved caspase-3 and Cleaved caspase-9 expression, alleviating sepsis-induced cell apoptosis of liver tissues (Figure 3(b)).

### PQQ repressed CUL3 expression in Kupffer cells suffered from LPS

**Figure 4.**
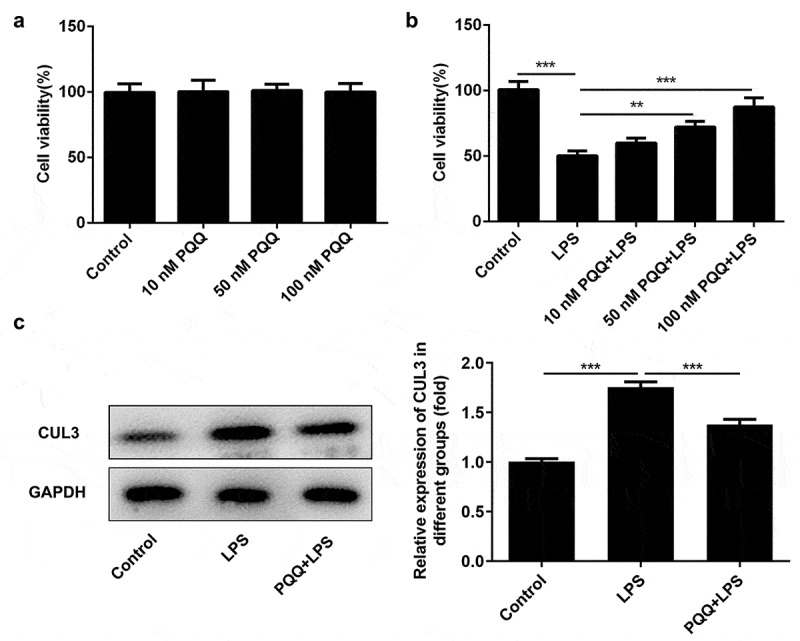
PQQ repressed CUL3 expression in Kupffer cells suffered from LPS. (a) Kupffer cells received treatment with PQQ (10, 50 and 100 nmol/L). Viability of Kupffer cells was measured by CCK-8 assay. (b) Kupffer cells were pretreated with PQQ (10, 50 and 100 nmol/L) for 2 h and then treated with 100 ng/mL LPS for 24 h. Viability of Kupffer cells was measured by CCK-8 assay. (c) CUL3 expression was determined using western blotting analysis

Kupffer cells were pretreated with PQQ (10, 50 and 100 nmol/L) for 2 h and then treated with 100 ng/mL LPS for 24 h, simulating sepsis-induced acute liver injury in vitro. Diverse concentration of PQQ showed no obvious influence on the viability of Kupffer cells (Figure 4(a)) while PQQ treatment could rescue the reduced viability of Kupffer cells suffered from LPS (Figure 4(b)). In addition, LPS stimulation enhanced CUL3 expression in Kupffer cells, which was partly abolished by PQQ therapy. This phenomenon prompted the potential role of CUL3 in the progression of sepsis (Figure 4(c)).

### Upregulation of CUL3 weakened the remission effects of PQQ on LPS-induced inflammatory and oxidative damage in Kupffer cells

**Figure 5.**
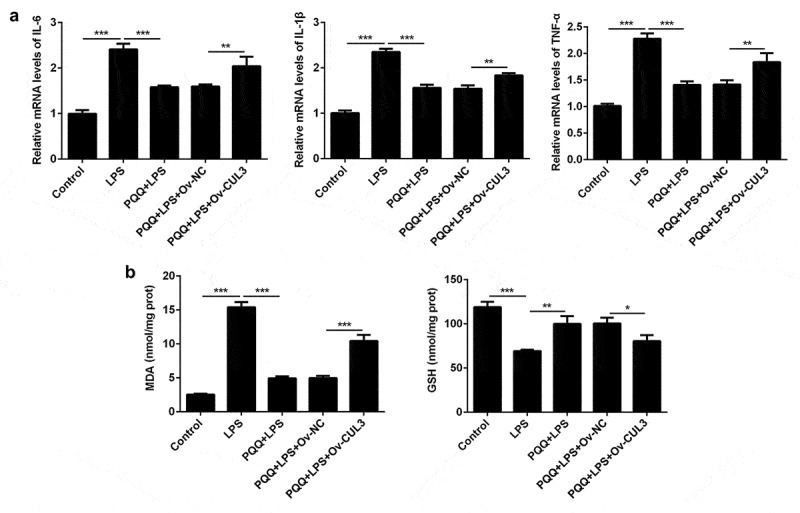
Upregulation of CUL3 weakened the remission effects of PQQ on LPS-induced inflammatory and oxidative damage in Kupffer cells. (a) Levels of IL-6, IL-1β and TNF-α in Kupffer cells were determined by RT-qPCR assay. (b) The production of MDA and GSH in Kupffer cells was detected with the commercial kits

To identify the underlying molecular mechanism, Kupffer cells were transfected with CUL3 overexpression vector. CUL3 expression was distinctly upregulated following transfection. Levels of IL-6, IL-1β and TNF-α were decreased after PQQ therapy and expressions of IL-6, IL-1β and TNF-α were enhanced again following CUL3 overexpression (Figure 5(a)). Similarly, the secretion of MDA was suppressed and GSH expression was enhanced after the treatment of PQQ, which was partly rescued by upregulation of CUL3 (Figure 5(b)). To sum up, PQQ may alleviate inflammatory and oxidative damage in LPS-induced Kupffer cells by downregulating CUL3 expression.

### Upregulation of CUL3 suppressed the remission effect of PQQ on LPS-induced apoptosis of Kupffer cells

**Figure 6.**
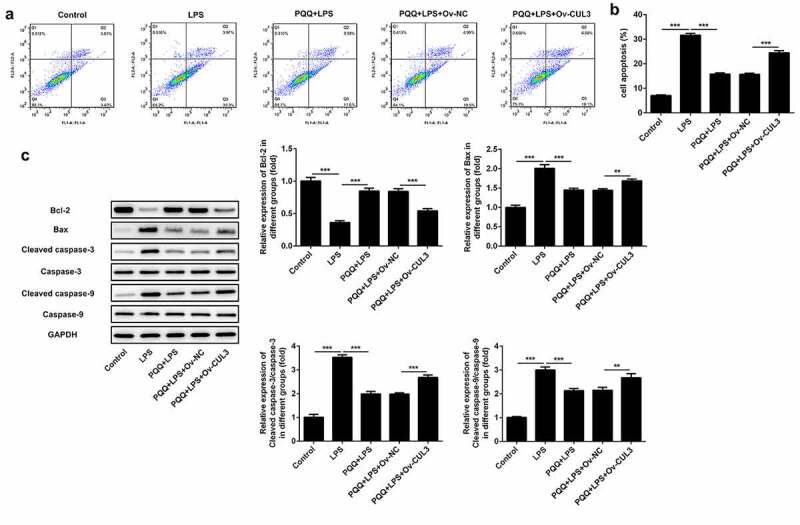
Upregulation of CUL3 suppressed the remission effect of PQQ on LPS-induced apoptosis of Kupffer cells. (a, b) Apoptosis rates of Kupffer cells were determined by flow cytometry analysis. (c) Expressions of Bcl-2, Bax, Cleaved caspase3 and Cleaved caspase9 were determined using western blotting analysis

In this part, it was confirmed that PQQ therapy reduced the apoptosis of Kupffer cells suffered from LPS. Moreover, the protective effect of PQQ on the apoptosis of LPS-induced Kupffer cells was partly abolished by upregulation of CUL3 (Figure 6(a, b)). In addition, PQQ treatment promoted Bcl-2 expression and suppressed the expressions of Bax, Cleaved caspase-3 and Cleaved caspase-9, and the regulating effects of PQQ on the expressions of apoptosis-related proteins were rescued following CUL3 overexpression (Figure 6(c)).

## Discussion

Sepsis is a systemic inflammatory response syndrome caused by bacterial or viral infections [18]. The occurrence of sepsis is usually accompanied by the development of metabolic disorder syndrome [19]. More importantly, sepsis often results in severe liver injury [20]. Normal liver function is the key to the survival of patients with sepsis. The dysregulation of inflammatory factors and liver damage induced by oxidative stress can also cause poor prognosis of patients with sepsis [21,22].

PQQ is a drug composed of nicotinamide and flavonoids. PQQ can improve the symptoms of osteoarthritis by alleviating oxidative damage, DNA damage and cell senescence of chondrocytes [10]. PQQ can also mitigate oxidative stress in diabetic mice [23]. Meanwhile, it is discovered that PQQ can relieve the symptoms of inflammatory response [11,24]. Moreover, PQQ could alleviate high glucose induced inflammation and senescence of HK-2 cells by restricting the production of ROS and suppressing Keap1 expression [15]. In this study, we found that PQQ treatment relieved liver damage induced by sepsis. Besides, PQQ lead to decreases in the levels of IL-1β, IL-6, TNF-α and MDA and an increase in GSH level. Apoptosis of liver tissue cells was alleviated after PQQ therapy. All these results above together evidenced that PQQ could mitigate sepsis-induced acute liver injury by alleviating inflammatory and oxidative stress damage and apoptosis of liver tissue cells.

There is a study that reveals that the levels of CUL3 are enhanced in LPS-treated mouse podocytes [16]. Meanwhile, Shufeng Jiedu capsule could reduce the levels of inflammatory cytokines and upregulate GST and SOD to protect the rat lungs suffered from LPS by suppressing Keap 1 and CUL3 [17]. To conclude, CUL3 may act as the promoting factor during the development of sepsis. In our current research, LPS stimulation also elevated CUL3 expression in Kupffer cells. In addition, we found that Keap1 has the potential to interact with CUL3 by querying the database. Furthermore, a study uncovers that increased interactions of Keap1 with CUL3 could promote renal fibrosis by reducing Nrf2 level [25]. Dayalan et al. [26] verifies that Keap1 is a substrate adaptor for a CUL3-based E3 ubiquitin ligase that regulates redox balance and inflammation. Then, it was confirmed in the present work that PQQ therapy rescued the increase of CUL3 expression in LPS-induced Kupffer cells. Overexpression of CUL3 partly abolished the protective effects of PQQ against inflammation, oxidative stress and cell apoptosis in Kupffer cells suffered from LPS.

## Conclusion

Overall, this current work identified the effects of PQQ on sepsis-induced acute liver injury and clearly expound the relevant molecular mechanism. In vivo and in vitro results strongly supported that PQQ relieved sepsis-induced acute liver injury, inflammatory and oxidative stress damage and cell apoptosis by repressing CUL3 expression.

## Data Availability

The datasets supporting the conclusions of this article are available from the corresponding author on reasonable request.
